# Endophytic bacterial community of a Mediterranean marine angiosperm (*Posidonia oceanica*)

**DOI:** 10.3389/fmicb.2012.00342

**Published:** 2012-09-21

**Authors:** Neus Garcias-Bonet, Jesus M. Arrieta, Charles N. de Santana, Carlos M. Duarte, Núria Marbà

**Affiliations:** ^1^Department of Global Change Research, IMEDEA (CSIC-UIB)Esporles, Spain; ^2^Oceans Institute, University of Western AustraliaCrawley, Australia

**Keywords:** *Posidonia oceanica*, seagrass-bacteria interaction, DGGE, endophytes

## Abstract

Bacterial endophytes are crucial for the survival of many terrestrial plants, but little is known about the presence and importance of bacterial endophytes of marine plants. We conducted a survey of the endophytic bacterial community of the long-living Mediterranean marine angiosperm *Posidonia oceanica* in surface-sterilized tissues (roots, rhizomes, and leaves) by Denaturing Gradient Gel Electrophoresis (DGGE). A total of 26 *Posidonia oceanica* meadows around the Balearic Islands were sampled, and the band patterns obtained for each meadow were compared for the three sampled tissues. Endophytic bacterial sequences were detected in most of the samples analyzed. A total of 34 OTUs (Operational Taxonomic Units) were detected. The main OTUs of endophytic bacteria present in *P. oceanica* tissues belonged primarily to Proteobacteria (α, γ, and δ subclasses) and Bacteroidetes. The OTUs found in roots significantly differed from those of rhizomes and leaves. Moreover, some OTUs were found to be associated to each type of tissue. Bipartite network analysis revealed differences in the bacterial endophyte communities present on different islands. The results of this study provide a pioneering step toward the characterization of the endophytic bacterial community associated with tissues of a marine angiosperm and reveal the presence of bacterial endophytes that differed among locations and tissue types.

## Introduction

Bacteria are commonly found living endophytically within plant tissues (e.g., Hallmann and Berg, 2006). Endophytic bacteria, typically defined as those living inside plant tissues not harming the host plant (Schulz and Boyle, 2006), often promote plant growth by, for instance, providing nutrients or controlling plant pathogens through mutualistic bacteria–plant interactions (e.g., Hallmann and Berg, 2006; Ikeda et al., 2010; Li et al., 2011). Moreover, symbionts can also be pathogenic bacteria that, when present at high abundances, cause plant disease outbreaks. Information on composition and ecological roles of symbiotic bacterial communities abounds for terrestrial and freshwater plants, particularly for crop species (e.g., Ueda et al., 1995); however, the presence and relevance of symbiotic bacterial communities in marine plants remain unexplored.

Seagrasses are marine clonal angiosperms that evolved from freshwater angiosperm ancestors that colonized the marine environment in the Cretaceous (den Hartog, 1970). Despite the fact that seagrass flora is restricted to approximately 50–60 species, they develop lush and highly productive meadows, particularly in oligotrophic waters, along the coasts of all continents except Antarctica (Hemminga and Duarte, 2000; Short et al., 2007). Seagrass meadows are important global carbon sinks, enhance coastal biodiversity and prevent coastal erosion (Hemminga and Duarte, 2000; Orth et al., 2006). Bacterial communities play important roles in seagrass meadows, particularly in the recycling of materials (Hemminga and Duarte, 2000). However, information about bacterial communities associated with seagrasses is scant, with most studies focusing on bacterial communities in seagrass sediments (Cifuentes et al., 2000; Bagwell et al., 2002; Garcia-Martinez et al., 2009) or associated with plant surfaces (i.e., epiphytic bacterial community) above (Weidner et al., 2000; Jensen et al., 2007; Uku et al., 2007; Crump and Koch, 2008) or belowground (Garcia-Martinez et al., 2005). However, endophytic bacteria in seagrass (*Thalassia hemprichii, Cymodocea serrulata, Halodule uninervis, Syringodium isoetofolium*) tissues have been reported using optical microscopy (Kuo, 1993). *Clostridium glycolicum* has been isolated from the rhizoplane and deep cortex cells of *Halodule wrightii* (Küsel et al., 1999), a new species of the genus *Sulfitobacter* has been isolated from a homogenate of *Zostera marina* (Ivanova et al., 2004), and *Desulfovibrio zosterae* has been isolated from the surface-sterilized roots of *Z. marina* (Nielsen et al., 1999), indicating that endophytic bacteria occur in seagrass tissues.

*Posidonia oceanica* is the dominant seagrass species in the Mediterranean Sea. Although *P. oceanica* ranks among the slowest growing seagrasses (rhizome extension rates ranging from 1 to 6 cm yr^−1^ apex^−1^, Marbà and Duarte, 1998), it develops meadows living for millennia (Mateo et al., 1997; Arnaud-Haond et al., 2012) and occupies an estimated 50,000 km^2^ in the Mediterranean Sea. The unique environments found in and around *P. oceanica* tissues constitute niches well differentiated from those in surrounding waters and sediments. Moreover, the millenary life span of *P. oceanica* clones suggest that endophytic bacteria can remain isolated within the *P. oceanica* tissues over extended periods of time, relevant for microbial evolutionary processes. Thus, it is likely that *P. oceanica* meadows harbor a distinct microbial community including previously undescribed species. Indeed, recent studies using culturing methods have described seven new bacterial species belonging to the genus *Marinomonas* isolated from *P. oceanica* (Espinosa et al., 2010; Lucas-Elío et al., 2011), supporting the idea of the existence of a distinct bacterial community associated with *P. oceanica*.

The interest in exploring the endophytic bacterial community of *P. oceanica* extends beyond that of exploring a potential biodiversity niche. The characterization of the microbes found inside the tissues of *P. oceanica* can offer significant clues about the health and ecology of *P. oceanica* meadows. Moreover, the number of disease outbreaks in the marine environment appears to be rising (Harvell et al., 1999). This trend is possibly facilitated by anthropogenic pressures (e.g., global movement of ballast waters by ships, Ruiz et al., 2000) and global warming (Harvell et al., 1999, 2002) as they may facilitate the occurrence of pathogens in areas with previously unexposed host populations. Symbiotic microorganisms can also play a key role in determining seagrass population dynamics as they can facilitate the uptake of elements like nitrogen, which can be limited in marine environments. The role of bacteria in sulfur cycling can also determine the health, and therefore the growth rates of marine angiosperms in marine sediments receiving high organic matter inputs. H_2_S produced from decomposition of organic matter under anoxic conditions can intrude into seagrass tissues (Pedersen et al., 2004), with negative consequences for seagrass meristematic activity (Garcias-Bonet et al., 2008). Bacteria can, therefore, play a major role in the survival and growth of seagrass meadows. The characterization of the microbiota closely associated with *Posidonia oceanica*, such as endophytic bacteria, is a first step that may provide further insights into the complex interactions between bacteria and seagrass.

Here we describe the bacterial communities associated with surface-sterilized tissues (roots, rhizomes, leaves) collected in summer in 26 meadows of *Posidonia oceanica* around the 950 km of coast of the Balearic Islands (Western Mediterranean). We used DGGE (Denaturing Gradient Gel Electrophoresis) to analyze the community structure of endophytic bacteria in the plant tissues. The banding profiles derived were compared across locations, and dominant bands were sequenced to provide a first identification of bacterial endophytes of Balearic *P. oceanica*.

## Materials and methods

### Sampling strategies

**Figure 1:**
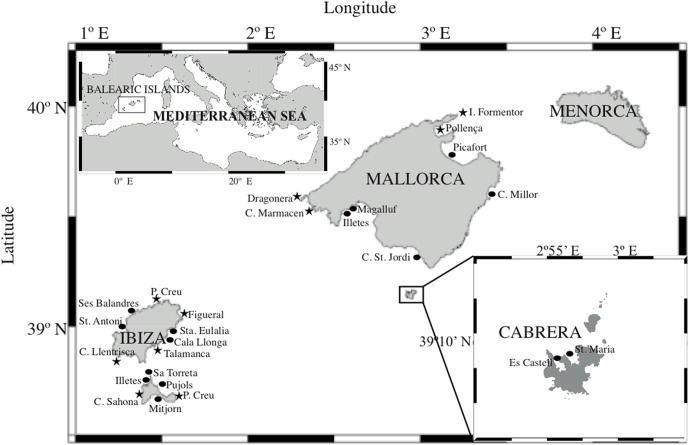
**Location of *P. oceanica* meadows sampled in summer 2005 (circles) and summer 2006 (stars)**.

*Posidonia oceanica* shoots were collected at 26 locations across the Balearic Islands (Figure 1) by SCUBA diving during the summers of 2005 and 2006. The plants were transported to the laboratory in seawater from the same location and processed immediately. The leaves, rhizomes, and roots from three shoots per meadow were separated and subsequently subjected to a surface-sterilization protocol adapted from Coombs and Franco (2003). Briefly, the protocol consisted of immersing each sample in 99% ethanol for 1 min, then in 3.125% NaOCl for 6 min, then in 99% ethanol for 30 s and finally washing gently with autoclaved seawater. These surface-sterilized samples were frozen in liquid nitrogen until nucleic acid extraction was performed.

### Nucleic acid extraction and amplification

Surface-sterilized plant material (100 mg of fresh tissue) was ground with the help of a sterilized pestle. The total nucleic acid extraction was performed using a commercial kit specific for plant tissues (Partec®). Nucleic acid extracts were stored at −20°C until amplification. The DNA extract, containing plant and endophyte DNA when present, was amplified by standard PCR with primers 907R (5′-CCG TCA ATT CCT TTG AGT TT-3′) and 341F-GC containing a 40 bp GC clamp at the 5′ end (5′-CGC CCG CCG CGC CCC GCG CCC GGC CCG CCG CCC CCG CCC C/CC TAC GGG AGG GAG CAG-3′) specific for the bacteria domain (Muyzer and Smalla, 1998). Additional negative (no DNA) and positive (*E. coli* DNA) control reactions were run with each batch of PCR reactions. The PCR products were checked by electrophoresis on 1% agarose gels. For each sample, the products of several replicate reactions (minimum of 2) were pooled prior to DGGE.

### Denaturing gradient gel electrophoresis (DGGE)

The amplification products of the fragment of the 16S ribosomal RNA gene (1 μg of PCR product) were separated by DGGE in a 6% polyacrylamide gel containing a gradient of denaturants ranging from 40 to 70% (where 100% is 7 M urea and 40% formamide). Gels were run for 18 h at 150 V in 1X TAE (Tris-Acetate-EDTA) buffer at 60°C in a CBS Scientific Co., DGGE system. Following electrophoresis, the gels were stained with SybrGold for 30 min in the dark and photographed using a G:BOX imaging system (Syngene). All the detectable bands were excised and stored frozen in autoclaved MiliQ water at −20°C for further processing.

### Analysis of DGGE profiles

The digital images of DGGE gels were analyzed by measuring the relative migration of each band, normalized to the migration of the 16S rDNA band corresponding to *Posidonia oceanica* chloroplasts, which were detectable on every sample. Additional DGGE gels containing replicates of PCR products already analyzed in different gels were run in order to facilitate comparisons across different DGGE gels. The bands with the same normalized migration distance were identified as the same Operational Taxonomic Unit (OTU), confirmed by sequencing of some bands.

Species accumulation curves (i.e., accumulated increase of the number of detected OTUs vs. number of samples) were constructed in R (R Development Core Team, 2011; http://www.R-project.org/) using package vegan (Oksanen et al., 2011) in order to check accuracy and representativeness of the sampling strategy and, therefore, of our results. Estimates of species richness (Chao, Jackknife, and Bootstrap) were obtained from accumulation curves using the function *specaccum* in package vegan.

A binary matrix (presence/absence) was constructed for all of the identified OTUs in order to determine the similarity among samples and locations. Using the information about presence/absence of each OTU in different tissues of *P. oceanica*, we have constructed weighted bipartite networks for each location studied to represent the endophyte–plant network of bacterial endophytes and *P. oceanica* tissues. In a bipartite network, there are nodes of two distinct types, and the edges connect only nodes of different kinds (Albert and Barabási, 2002; Newman, 2003). For the networks used in this study, one set of nodes was composed of the 34 detected OTUs, and the other set was composed of the tissue groups (roots, rhizomes, and leaves), totaling 37 nodes. Links between the two sets of nodes were drawn if an OTU was observed in a tissue, and the weights of these links were represented by the sum of the relative observations of each OTU. We call “relative observation of an OTU” the ratio between the number of observations of the OTU in the tissue and the number of replicates in each location studied.

Once the weighted bipartite networks for each location were constructed, we collapsed the networks of locations at the same island (Cabrera, Formentera, Ibiza, and Mallorca) in order to obtain the weighted bipartite network for each island. By collapsing the networks of all locations, we obtained the weighted bipartite network for the Balearic Archipelago.

We compared the bipartite networks of each island using the concept of the distance between networks with the same number of nodes, as described by Andrade et al. (2008). They used the shortest paths and the diameter of a pair of networks to give a quantitative and normalized value to represent the similarity between these networks. We adapted their method by using the weighted shortest path and weighted diameter in order to compare the similarity in the weighted bipartite network among islands.

We used a bootstrap strategy to examine the robustness of the network analysis. We randomly removed one node of the networks to be compared and computed the distance between them. After repeating this procedure for each node, we calculated the average of the distances computed for each pair of networks. This average was considered as the best estimate of the average distance between any pair of networks, and the procedure was repeated for each pair of networks (each pair of locations).

We used the Girvan–Newman algorithm (Girvan and Newman, 2002) to identify which nodes of the Balearic Island bipartite network were more densely grouped representing communities of endophytic microorganisms that tended to co-occur. Although the original Girvan–Newman algorithm was developed for unweighted, undirected networks, here we have adapted this algorithm to enable the community analysis of weighted networks, as suggested by Yoon et al. (2006).

The binary matrix was also used to generate a distance matrix based on Jaccard's coefficient as the basis for a non-metric multidimensional scaling (NMDS) diagram using package vegan in R. We performed an Analysis of Similarity (ANOSIM) using the vegan package (10,000 permutations), to test for the existence of differences in band patterns among tissue groups defined as roots, rhizomes, and leaves. The R value generated by ANOSIM test indicates the magnitude of difference among groups, where an *R* > 0 indicates differences between groups and *R* < 0 indicates no difference between groups, because differences between groups are lower than differences within a group. The significance of ANOSIM results was tested using the Bonferroni correction as *post-hoc* test.

Finally, we performed an indicator species test (Dufrene and Legendre, 1997) using package labdsv (Roberts, 2010) in R software to identify those OTUs that are characteristics of each tissue and island. The indicator species are defined as the most characteristic species of each group, found mostly in a single group and present in the majority of the sites or samples belonging to that group.

### Sequencing of the OTUs detected in DGGE

The detected and excised bands (OTUs) from the DGGEs were reamplified using the same pair of primers (907R and 341F-GC). The amplification products were cleaned and purified from primers and dNTPs by an enzymatic reaction with a mixture of Exonuclease I (1 U/reaction) and Alkaline Phosphatase (1 U/reaction) at 37°C during 60 min, followed by an enzyme denaturing step at 72°C for 15 min. The DNA was precipitated using isopropanol (66% final concentration), centrifuged (10,000× g, 15 min), washed with 66% isopropanol and resuspended in sterile water. The resulting DNA concentration was measured fluorometrically (Qubit®, Invitrogen) and 150 ng of the amplified product was used for the sequencing reaction using the reverse primer 907R. The sequencing was performed by Secugen, using the chemistry BigDye® Terminator v3.1. The sequences of about 500 bp were checked for existence of chimeras using the Bellerophon tool available at http://greengenes.lbl.gov and compared to the public DNA database of NCBI by using BLAST (Basic Local Alignment Search Tool) service at the National Center of Biotechnology Information (NCBI) web page (www.ncbi.nlm.nih.gov). Further validation of the phylogenic identity of the sequences was performed by aligning the sequences to those in the greengenes database (http://greengenes.lbl.gov) using ARB (Ludwig et al., 2004). A Neighbor Joining tree of full sequences of the closest relatives was constructed in ARB and the shorter DGGE sequences were added to that tree using the ARB parsimony interactive tool. Bootstrap values were also generated using the ARB interactive parsimony tool.

The sequences obtained in this study have been deposited in Genbank under the accession numbers JF292432 to JF292446.

## Results

A total of 34 different OTUs were identified in DGGE profiles from all plant tissue samples (*n* = 186). Rhizome samples (*n* = 57) and root samples (*n* = 67) hosted 28 different OTUs while leaf samples (*n* = 62) showed 24 different OTUs detectable by DGGE analysis. Thirteen rhizome samples (18.6%), five root samples (6.9%), and eight leaf samples (11.4%) did not show any band, except the band corresponding to the 16S rDNA of the chloroplast.

**Figure 2:**
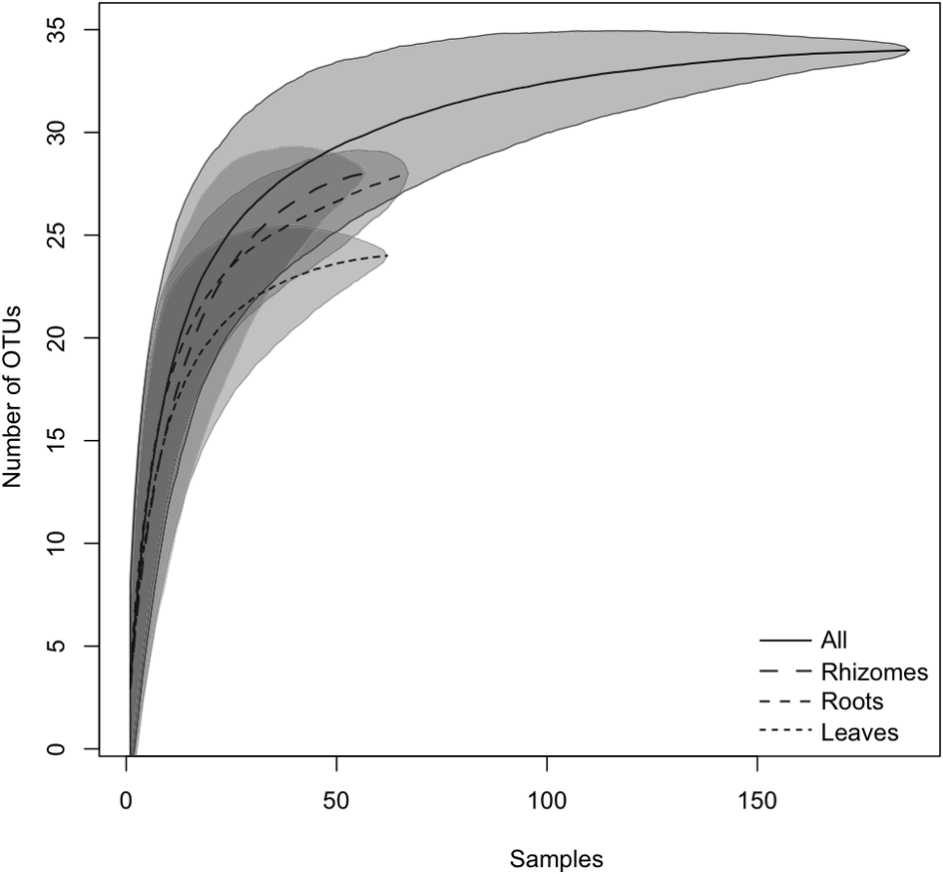
**Species accumulation curves of the endophytic bacterial community found in *P. oceanica* tissues.** The lines indicate the averaged accumulated increase of detected OTUs vs. number of samples (10,000 bootstrap sampling replicates). The shadowed area indicates the standard deviation. The continuous line represents all the samples pooled together, and the dashed lines provide the values for the different tissues.

The species accumulation curves (Figure 2) confirmed that the sampling effort was adequate to characterize the bacterial community richness associated with *P. oceanica* tissues, as curves showed saturation (i.e., approached a plateau), suggesting that more intensive sampling was likely to yield only minor improvements in coverage. The Chao, Jackknife, and Bootstrapping estimates of species richness (Table 1) indicated that the percentages of OTUs detected in our DGGE gels accounted for 97–99.6% of the total community richness for all tissues sampled. Large coverage was estimated for all tissue classes, the percentages of detected richness varied between 95.6–99.6% (leaves), 93.4%–98.9% (rhizomes) and from 69.1 to 93.1% (roots) depending on the particular estimate used. Despite the relatively high numbers of OTUs found overall, individual samples of different tissues contained relatively low numbers of OTUs. Roots presented 3.56 ± 2.5 different OTUs (average ± SD) per sample, while only 2.46 ± 2.4 and 2.7 ± 2.5 OTUs were found in rhizomes and leaves, respectively.

**Table 1:** **Number of total OTUs detected in each tissue for all the samples tested; total number of OTUs estimated by different approaches: Chao, Jackknife and Bootstrapping; and percentage of the total estimated OTUs detected in the samples**.

			**Number of OTUs expected by**			**Percentage of sampled OTUs from the expected number by**		
**Groups**	**N**	**Number of OTUs detected**	**Chao approach**	**Jackknife approach**	**Bootstrapping approach**	**Chao approach**	**Jackknife approach**	**Bootstrapping approach**
All	186	34	34.12 ± 0.44	34.99 ± 0.99	35.04 ± 0.98	99.65	97.16	97.03
Leaves	62	24	24.1 ± 0.38	24.98 ± 0.98	25.09 ± 1.28	99.59	96.06	95.64
Rhizomes	57	28	28.29 ± 0.68	29.96 ± 1.38	29.89 ± 1.67	98.97	93.44	93.67
Roots	67	28	40.5 ± 17.14	32.93 ± 2.61	30.08 ± 1.34	69.14	85.04	93.08

N = number of samples.

**Figure 3:**
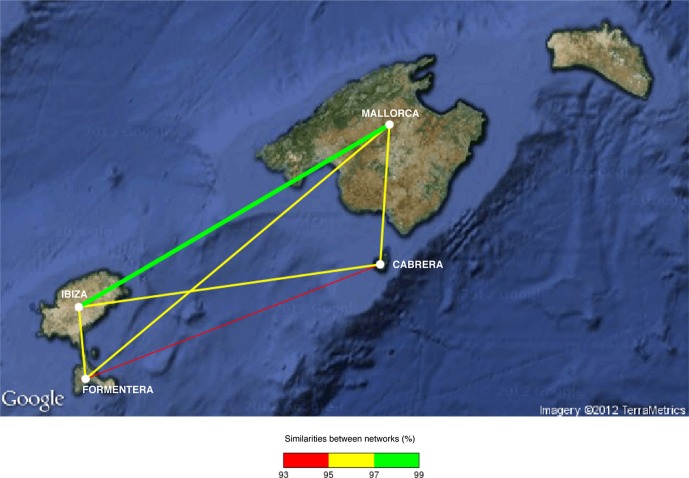
**Bipartite network analysis of endophytic bacterial community of *P. oceanica* tissues among islands.** The nodes represent the islands. Thicker edges between two islands indicate larger similarity of the bipartite networks. Edges are also color-coded to indicate the percentage of similarity between two islands.

The bipartite network analysis showed differences in the band patterns among islands (Figure 3), where meadows located in Ibiza and Mallorca islands seemed to be the most similar among them (more than 97% of similarity). Conversely, the networks of Cabrera and Formentera islands were the most different (93–95% of similarity). The other pairs of islands (Ibiza–Formentera; Ibiza–Cabrera; Mallorca–Cabrera; Mallorca–Formentera) have endophyte–plant networks with 95–97% similarity among them.

**Figure 4:**
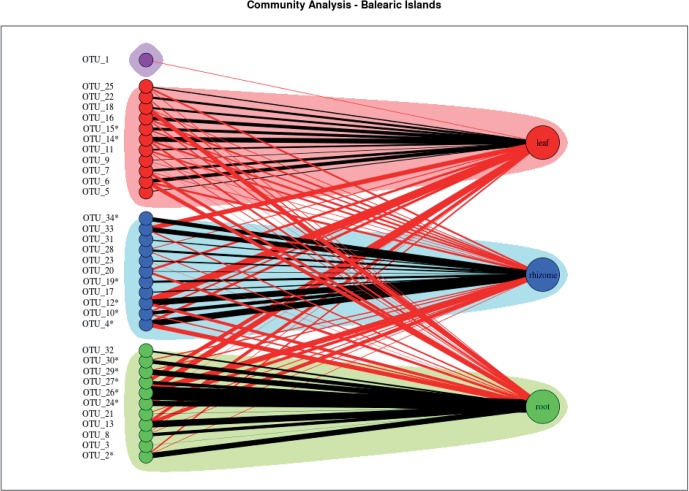
**Community analysis of the bipartite network of all the Balearic Islands studied.** Shadowed areas indicate communities strongly associated with leaf (red), rhizome (blue), and root (green). The node representing the OTU 1 was not associated to any community and is shown in purple. Black edges connect nodes at same community, and red edges connect nodes at different communities. The widths of the edges connecting OTUs to tissues are proportional to the total number of observations of each OTU in each tissue divided by the number of replicates. ^*^ = Statistically significant.

The community analysis of the bipartite network of all the Balearic Islands studied, obtained by running the Girvan–Newman algorithm, and identified three different communities for each tissue type (Figure 4). The algorithm did not associate any community with the OTU 1.

Although NMDS did not show clear differences among tissues (data not shown), ANOSIM test confirmed that band patterns among tissues were different with statistically significance, although these differences were small, suggesting other variables play a role in the endophytic bacterial composition of *P. oceanica* tissues. The band patterns obtained in DGGE analysis for root tissues were different from those obtained for rhizome and leaf tissues (*R* = 0.201, *P* < 0.005 and *R* = 0.126, *P* < 0.005, respectively) and greater than the differences obtained between band patterns in leaf and rhizome tissues (*R* = 0.046, *P* < 0.05).

Moreover, the indicator species analysis identified some OTUs characteristic of each tissue (Table 2), although the indicator values were low. According to indicator species analysis, two OTUs were associated with leaves, five OTUs were associated with rhizomes, and six OTUs were associated with roots.

**Table 2:** **Indicator species test**.

	**Times of appearance in**						
**OTUs**	**Roots**	**Rhizomes**	**Leaves**	**All**	**Cluster**	**Indicator value**	**Probability**
**OTU_15**	**1**	**4**	**12**	**17**	**Leaves**	**0.1344**	**0.001**
**OTU_14**	**7**	**5**	**17**	**29**	**Leaves**	**0.1612**	**0.004**
OTU_33	5	9	13	27	Leaves	0.0994	0.186
OTU_1	0	0	2	2	Leaves	0.0323	0.209
OTU_5	0	0	2	2	Leaves	0.0323	0.216
OTU_7	6	1	7	14	Leaves	0.0579	0.232
OTU_16	5	5	8	18	Leaves	0.0571	0.482
OTU_6	12	7	12	31	Leaves	0.0756	0.63
OTU_11	3	5	6	14	Leaves	0.0408	0.698
**OTU_34**	**0**	**8**	**0**	**8**	**Rhizomes**	**0.1404**	**0.001**
**OTU_4**	**15**	**21**	**6**	**42**	**Rhizomes**	**0.197**	**0.005**
**OTU_19**	**0**	**4**	**0**	**4**	**Rhizomes**	**0.0702**	**0.011**
**OTU_10**	**5**	**13**	**6**	**24**	**Rhizomes**	**0.1302**	**0.019**
**OTU_12**	**10**	**26**	**25**	**61**	**Rhizomes**	**0.2063**	**0.019**
OTU_31	0	2	0	2	Rhizomes	0.0351	0.074
OTU_23	1	3	0	4	Rhizomes	0.041	0.079
OTU_17	0	2	0	2	Rhizomes	0.0351	0.095
OTU_21	1	2	0	3	Rhizomes	0.0246	0.199
OTU_25	2	3	2	7	Rhizomes	0.0241	0.675
OTU_9	1	2	2	5	Rhizomes	0.015	0.875
**OTU_26**	**39**	**13**	**21**	**73**	**Roots**	**0.2949**	**0.001**
**OTU_29**	**24**	**2**	**0**	**26**	**Roots**	**0.3263**	**0.001**
**OTU_30**	**13**	**2**	**0**	**15**	**Roots**	**0.1643**	**0.001**
**OTU_27**	**14**	**0**	**8**	**22**	**Roots**	**0.1292**	**0.004**
**OTU_2**	**14**	**1**	**5**	**20**	**Roots**	**0.1422**	**0.005**
**OTU_24**	**17**	**8**	**1**	**26**	**Roots**	**0.1569**	**0.006**
OTU_8	7	2	2	11	Roots	0.0635	0.104
OTU_32	3	0	0	3	Roots	0.0448	0.136
OTU_18	11	3	7	21	Roots	0.0818	0.157
OTU_20	11	3	9	23	Roots	0.0745	0.288
OTU_22	5	0	4	9	Roots	0.04	0.289
OTU_13	18	12	11	41	Roots	0.1099	0.405
OTU_28	5	4	3	12	Roots	0.0288	0.921
OTU_3	1	0	0	1	Roots	0.0149	1

Number of times each OTU was found in each tissue class, and in total. Cluster indicates the group for which each OTU is a likely indicator, with the indicator value and the associated probability. Statistically significant indicator species shown in bold.

**Figure 5:**
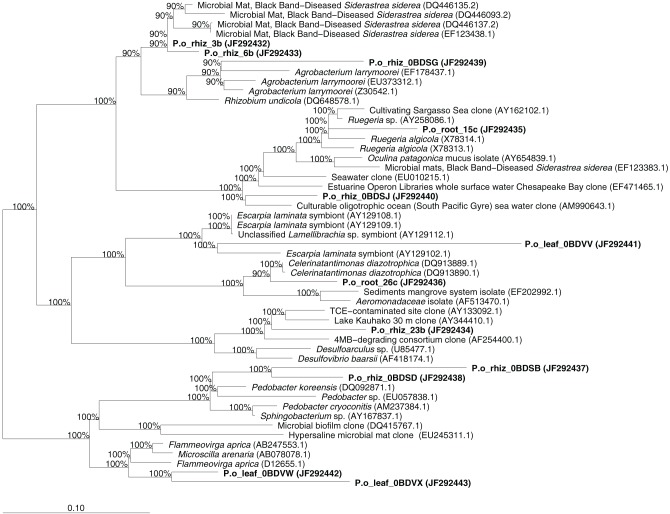
**Phylogenetic affiliation of the sequences obtained from the endophytic bacterial community of *P. oceanica*.** Sequences obtained in this study are printed in bold. The values at each node represent the bootstrap values generated using the ARB parsimony interactive tool.

We sequenced approximately 200 bands detected by DGGE analysis, trying to cover all identified OTUs. However, we only managed to obtain 12 different bacterial sequences. Totally 33.3% of the sequences analyzed belonged to Bacteroidetes, while the rest (66.7%) belonged to the class Proteobacteria: 41.7% were affiliated to the α-subclass, 16.7% to the γ-subclass, and 8.3% to the δ-subclass. More specifically, 15.4% of the sequences belonged to the *Desulfovibrionaceae*, 15.4% to the *Flammeovirgaceae*, 15.4% to the *Rhodobacteracerae*, 15.4% were *Sphingobacteriaceae*, 15.4% non-identified Coral Black Band Disease isolates, 7.7% *Oceanimonaceae*, 7.7% *Rhizobiaceae*, and 7.7% were non-identified Sulfur-Oxidizing Symbionts. We identified three endophytic bacteria characteristic of leaf tissues, seven of rhizome tissues, and two of roots tissues. The closest relative sequences to our OTUs are listed in Table A1 and the phylogenetic assignment is illustrated in Figure 5.

## Discussion

The results reported here provide a pioneering step toward the characterization of the endophytic bacterial community associated with tissues of a marine angiosperm, by both comparing DGGE band patterns and sequencing the main OTUs found. Our results show that endophytic bacteria are frequently present in tissues of *P. oceanica* in the Balearic Islands, as most samples analyzed (93.1% of roots, 81.4% of rhizomes, and 88.6% of leaves) carried endophytic bacteria. However, a more exhaustive survey using larger amounts of tissue and/or other techniques more sensitive to low amounts of DNA could yield even higher percentages of plants carrying bacterial endophytes.

Whereas our study appeared to yield a thorough inventory of OTUs in tissues of *P. oceanica*, the number of different OTUs identified in *P. oceanica* tissues, by DGGE analysis, appears relatively small, with 34 OTUs detected in *P. oceanica* tissues, which suggest that the endophytic microbiota must be highly specialized. The endophytic bacterial diversity reported here is similar to that found using comparable methodologies in other plants, such as rice (*Oryza sativa*), where 52 different endophytic OTUs were identified in a library of 192 clones. About 60% of endophytic OTUs detected on rice were Proteobacteria (Sun et al., 2008), similar to the percentage obtained for *P. oceanica* tissues. Similar numbers of epiphytic bacterial OTUs were obtained by DGGE in marine green *Ulvacean* algae where 34 sequences were reported, most of which belonged to Proteobacteria with minor representation of Bacteroidetes (Tujula et al., 2010). However, the endophytic bacterial community characterized by DGGE analysis in potato (*Solanum tuberosum*) and maize (*Zea mays*) plants was less diverse with 11 OTUs for potato plants (Garbeva et al., 2001) and six different bacterial species, all identified as proteobacteria, for maize roots (Seghers et al., 2004). When compared with other marine organisms, the bacterial diversity described in *P. oceanica* is similar to that found in the marine sponge *Rhopaloides odorabile*, where 34 different bacterial sequences were obtained from a clone library of 70 clones obtained from of three samples (Webster et al., 2001).

Our estimates of bacterial endophyte richness are influenced by the choice of DGGE in our survey. Much higher numbers of OTUs could be expected from large cloning efforts or from the use of massively parallel sequencing techniques. Webster et al. (2010) found 2996 different bacterial OTUs in sponges using 454 tag sequencing while the same sponges only yielded 34 sequences out of a library of 70 clones (Webster et al., 2001). Similarly, 1178 clones obtained from only 14 coral samples yielded 430 distinct bacterial ribotypes of endo- and epibionts by clone library techniques (Rohwer et al., 2002). Thus, DGGE or cloning of a limited number of clones can only detect the highly abundant members of the bacterial community and it is possible that use of high-throughput sequencing techniques would result in the detection of a much larger number of low abundance endophytes. Therefore, the number reported here is a minimum estimate of the species richness of the bacterial endophytes of *P. oceanica*.

In contrast, most of the ribotypes reported by deep-sequencing studies are present in a very low abundance with only one or a few sequences out of several thousands or more (Webster et al., 2010). From a functional point of view, it is likely that those microbial types present in high abundance possess a biomass high-enough to contribute significantly to the metabolism of the plant, while the contribution of rare bacterial ribotypes is probably less significant. Thus, our minimal estimate of the highly abundant ribotypes obtained by DGGE analysis is likely to represent those bacterial endophytes having a more profound impact on the biology of *P. oceanica.*

The comparison of patterns in endophytic bacterial communities between tissues suggested that bacteria associated with roots differ from those associated with rhizomes and leaves, similar to what was found among rice tissues (García de Salamone et al., 2010). This was later confirmed by the ANOSIM test and the community analysis of bipartite networks (Figure 4). *P. oceanica* tissues experience a different range of physical and chemical environmental conditions due to their nature, such as light and oxygen concentration gradient and also toxic metabolites found mainly in sediment. The specific environment where each tissue is located can select for bacterial species that can survive. The presence of toxic metabolites such as sulfide that can intrude plant tissues (Frederiksen et al., 2007) can be conditioning the bacterial species that can survive and develop in each tissue compartment. Moreover, some OTUs were identified as indicator species in roots, rhizomes, and leaves of *P. oceanica*, confirming the existence of a distinct endophytic community in each tissue. Differences in endophytic bacterial community patterns in *P. oceanica* due to the type of tissue were small, suggesting the importance of the meadow from which the tissue was collected and therefore, the importance of environmental factors affecting each location. In each island we can find a broad range of anthropogenic perturbation, with some locations being highly impacted by bathing and boat traffic and some others more pristine receiving fewer visitors per year. The bacterial community composition appeared to be related to the geographical location of the sampled meadows (Figure 3). Those meadows located in Mallorca and Ibiza, the two islands subjected to higher pressure from tourism were most similar as compared to Cabrera and Formentera, the more pristine, less visited islands. This suggests that anthropogenic perturbation may have an impact on the bacterial communities inhabiting *P. oceanica* tissues.

The sequencing of the main OTUs detected by DGGE analysis allowed us to draw the first identification of the endophytic bacterial community in *Posidonia oceanica* tissues. The main group represented is the Proteobacteria class comprising 66% of the OTUs, with the a-subclass being the majority group, as is characteristic for marine environments. The other main group is represented by Bacteroidetes, with many representatives found in marine environments. Most of the bacterial OTUs belonged to *Desulfovibrionaceae, Flammeovirgaceae, Rhodobacteracerae, Sphingobacteriaceae*, and Non-identified Coral Black Band Disease isolates. Less common groups were *Oceanimonaceae, Rhizobiaceae*, and Non-identified Sulfur-Oxidizing Symbionts.

The identification of bacteria similar in sequence to those found in diseased coral tissues opens a new and exciting research line, as there is no evidence, to our knowledge, of specific bacterial pathogens of seagrasses. However, demonstrating the pathogenicity of these organisms will require further research, involving the isolation of the potential causative agents and demonstrating that they fulfill Koch's postulates. Some of these bacteria found in diseased corals have been identified in association with macroalgae without relation to disease (Table A1) and others could be just opportunistic microbes degrading already damaged tissues. Some of the sequences were similar to those of sulfur-oxidizing symbionts (Figure 5). The presence of sulfur-oxidizing bacteria capable of oxidizing the sulfide to elemental sulfur would have an important role in detoxifying sediments because hydrogen sulfide produced as a consequence of organic matter decomposition is toxic for plants (Calleja et al., 2007; Garcias-Bonet et al., 2008). This would be particularly beneficial for *P. oceanica* survival in carbonate and iron poor sediments, characteristic of the Balearic coast and many other Mediterranean areas, where low iron available in these sediments prevents formation of iron sulfur compounds, and thus even small inputs of organic matter are able to enhance pore water hydrogen sulfide concentration (Holmer et al., 2003; Marbà et al., 2007).

Similarly, we identify bacteria similar to sequences found endophytically in other plants and related to *Rhizobiaceae* (Figure 5), with many species that are able to fix nitrogen in symbiosis with plants. The identification of bacteria related to well known nitrogen fixers is specially interesting because the Mediterranean sediments are known to be oligotrophic and the existence of bacteria with capabilities of shaping the nutrient conditions may have a beneficial role in the establishment, growth and survival of *P. oceanica* in this environment. This is particularly the case for bacteria belonging to the *Rhizobiaceae*, as *Agrobacterium* species are aerobic bacteria that can live free as well as some strains are responsible of tumor formation in terrestrial plants. In fact, there is a marine subdivision of *Agrobacterium* species (Uchino et al., 1997), although their role is still not clear. Moreover, PCR amplification of *nifH* genes from *P. oceanica* tissues confirmed the presence of diazotrophs (Garcias-Bonet et al., submitted).

In summary, this work is the first characterization of endophytic bacterial community in *Posidonia oceanica* tissues, suggesting the presence of specialized bacterial phylotypes in roots. The presence of bacterial endophytes in most of the samples analyzed indicates that these endophytes may be playing important roles in the physiology and survival of *P. oceanica* in the Mediterranean Sea. However, further research is needed to explain the different patterns observed across tissues and meadows. Moreover, this work represents the first identification of endophytic bacteria present in *P. oceanica* tissues. Some of the sequences were closely related to major groups of bacteria able to fix nitrogen, some others related to the sulfur cycle and finally a group of sequences had their closest known relatives among those found in diseased corals. It is not possible to infer whether or not the functional genes and capacities associated to the closest matching relatives will be present in our samples, due to the low similarity of some sequences to known cultured bacteria or even to environmental sequences. However, the fact that the closest matches are related to these three categories suggests that endophytic bacteria may play an important role in the health of *P. oceanica* by providing nitrogen and protecting the plants against the invasion of toxic sulfides. Moreover, the low sequence similarity to previously reported sequences in Genbank indicates that many of these sequences correspond to unknown bacteria, some of which could be specific to *P. oceanica* tissues. Subsequent research should include a search for functional genes involved in nitrogen fixation and the sulfur cycle and also a more detailed study on healthy vs. damaged tissues of *P. oceanica*, which could lead to the discovery of unknown bacterial pathogens of marine angiosperms.

### Conflict of interest statement

The authors declare that the research was conducted in the absence of any commercial or financial relationships that could be construed as a potential conflict of interest.

## Appendix

**Table A1:** **Closest relatives of the sequenced OTUs**.

**Sequence ID (accession number)**	**Tissue/location**	**Length (bp)**	**Similarity (%)**	**Closest relative in BLAST (accession number)**	**Description**	**Author**	**Family/group in greengens**
P.o_rhiz_3b (JF292432)	Rhizome/C.Sahona	494	99	Uncultured bacteria (EU181012)	Isolated from seagull fecal sample	Lu et al., 2008	Non-Identified Coral BBD isolates/α-proteobacteria
			99	Uncultured bacterium clone (GU118071)	Isolated from the coral *Acropora palmata*	Sunagawa et al., 2010	
			99	Uncultured bacterium clone (FJ202069)	Isolated from the coral *Montastraea faveolata* displaying signs of White Plague Disease type II	Sunagawa et al., 2009	
			99	Uncultured bacterium clone (EF123439)	Isolated from Black Band Diseased tissues of the coral *Siderastrea siderea*	Sekar et al., 2008	
			99	*Cohaesibacter* sp. (GQ200200)	Rhizobiales; Cohaesibacteraceae. Isolated from sediment of a seawater pond used for sea cucumber culture	Qu et al., 2011	
P.o_rhiz_6b (JF292433)	Rhizome/Figueral	433	97	Uncultured bacteria (EU181012)	Isolated from seagull fecal sample	Lu et al., 2008	Non-Identified Coral BBD isolates/α-proteobacteria
			97	Uncultured bacterium clone (GU118071)	Isolated from the coral *Acropora palmata*	Sunagawa et al., 2010	
			97	Uncultured bacterium clone (FJ202069)	Isolated from the coral *Montastraea faveolata* displaying signs of White Plague Disease type II	Sunagawa et al., 2009	
			97	Uncultured bacterium clone (EF123439)	Isolated from Black Band Diseased tissues of the coral *Siderastrea siderea*	Sekar et al., 2008	
			97	*Cohaesibacter* sp. (GQ200200)	Rhizobiales; Cohaesibacteraceae. Isolated from sediment of a seawater pond used for sea cucumber culture	Qu et al., 2011	
P.o_rhiz_23b (JF292434)	Rhizome/Talamanca	518	92	Uncultured δ-proteobacteria (AY133092)	Isolated from TCE-contaminated site	Carrol and Zinder, Unpublished	*Desulfovibrionaceae*/δ-proteobacteria
			92	Uncultured bacterium clone (GU118736)	Isolated from the coral *Montastraea franksi*	Sunagawa et al., 2010	
			91	Bacterium enrichment culture clone (HQ622261)	Polluted estuarine sediment	Abed et al., Unpublished	
			91	*Desulfarculus baarsii* (CP002085)	Desulfarculales; Desulfarculaceae; Desulfarculus	Lucas et al., Unpublished	
P.o_root_15c (JF292435)	Root/C.Marmacen	471	96	*Roseovarius sp.* (HQ871860 + HQ871851)	Rhodobacterales; Rhodobacteraceae; Roseovarius	Jeanthon et al., Unpublished	*Rhodobacteraceae*/α-proteobacteria
			96	*Pelagibaca sp.* (EU440959)	Rhodobacterales; Rhodobacteraceae; Pelagibaca	Yuan et al., Unpublished	
			96	Rhodobacterales bacterium (HQ537377 + HQ537273)	Isolated from 75 m depth on C-MORE BLOOMER cruise, Hawaii Ocean Time Series (HOT) station ALOHA“	Sher et al., Unpublished	
			96	Uncultured Rhodobacterales bacterium (GU474886)	Isolated from Hawaii Oceanographic Time-series study site ALOHA“	Rich et al., 2011	
			96	*Marinovum algicola* (FJ752526)	Rhodobacterales; Rhodobacteraceae; Marinovum	Pradella et al., 2010	
P.o_root_26c (JF292436)	Root/Magalluf	520	94	*Celerinatantimonas diazotrophica (DQ913889)*	Nitrogen Fixing Bacteria isolated from stuarine grasses *Spartina alterniflora* and *Juncus roemerianus*	Cramer et al., 2011	*Oceanimonaceae*/γ
			93	*Celeribacter arcticus* gen. nov., sp. nov. (FJ039852)	Alteromonadales. Halophilic denitrifying bacteria isolated from water brine in Siberian permafrost	Shcherbakova et al., Unpublished	
			92	Uncultured *Agarivorans sp.* (DQ647161)	Alteromonadales; Alteromonadaceae; Agarivorans	Dahle et al., 2008	
			92	*Agarivorans* sp. (GQ200591)	Alteromonadales; Alteromonadaceae; Agarivorans. Isolated from surface of seaweeds	Du et al., 2011	
P.o_rhiz_0BDSB (JF292437)	Rhizome/C.Torreta	391	91	Uncultured bacterium clone (GU946163)	Isolated from agricultural soil	Ros et al., Unpublished	*Sphingobacterriaceae*/ Bacteroidetes
			90	*Pedobacter sp.* (AM988948)	Bacteroidetes; Sphingobacteria; Sphingobacteriales; Sphingobacteriaceae; Pedobacter. Isolated from lake water	Berg et al., 2009	
			90	*Pedobacter koreensis* (DQ092871)	Bacteroidetes; Sphingobacteria; Sphingobacteriales; Sphingobacteriaceae; Pedobacter. Isolated from fresh water	Baik et al., 2007	
P.o_rhiz_0BDSD (JF292438)	Rhizome/C.Torreta	412	96	Uncultured bacterium clone (HM125351)	Isolated from soils	Bissett, Unpublished	*Sphingobacteriaceae*/ Bacteroidetes
			95	Uncultured bacterium (AM158409)	Isolated from *Typha* rhizosphere in constructed wetlands	Saenz de Miera et al., Unpublished	
			95	Bacterium (FJ654260)	Isolated from soil	Kim et al., Unpublished	
			95	Uncultured bacterium clone (GU946163)	Isolated from agricultural soil	Ros et al., Unpublished	
			95	*Pedobacter sp.* (HM204919)	Bacteroidetes; Sphingobacteria; Sphingobacteriales; Sphingobacteriaceae; Pedobacter	Im, Unpublished	
P.o_rhiz_0BDSG (JF292439)	Rhizome/C.Torreta	368	92	Unidentified bacterium clone (EF606109)	Isolated from rhizosphere soil from former arable field sown with low seeds diversity	Kielak et al., 2008	*Rhizobiaceae*/α-proteobacteria
			92	Uncultured bacterium clone (HM066499)	Isolated from environmental sample	Gray and Engel, Unpublished	
			92	Uncultured bacterium clone (AB583099)	Isolated from soybean leaf	Ikeda et al., 2011	
			92	*Agrobacetrium tumefaciens* (HQ003411)	Rhizobiales; Rhizobiaceae; Rhizobium/Agrobacterium group; Agrobacterium. Isolated from a lake	Sahay et al., Unpublished	
			92	*Agrobacetrium tumefaciens* (FJ999942)	Rhizobiales; Rhizobiaceae; Rhizobium/Agrobacterium group; Agrobacterium. Isolated from a plant, endophytic microbiota	Zheng and Feng, Unpublished	
P.o_rhiz_0BDSJ (JF292440)	Rhizome/Porto Colom	404	97	*Nautella italica* (HQ908722)	Rhodobacterales; Rhodobacteraceae; Nautella. Bacteria associated with sponges	Feby and Nair, Unpublished	*Rhodobacteraceae*/α-proteobacteria
			97	*Nautella* sp. (HQ188608)	Rhodobacterales; Rhodobacteraceae; Nautella. Isolated from surface seawater	Cho and Hwang, 2011	
			95	Uncultured bacterium clone (GU472165)	Isolated from BBD affected corals	Arotsker et al., Unpublished	
			97	*Ruegeria* sp. (GU176618)	Rhodobacterales; Rhodobacteraceae; Ruegeria. Isolated from surface of the red macroalgae, *Delisea pulchra*	Case and Kjelleberg, Unpublished	
			97	Rhodobacteraceae bacterium (FJ937900)	Rhodobacterales; Rhodobacteraceae. Isolated from *Gelliodes carnosa* (marine sponge)	Li et al., Unpublished	
			97	Uncultured bacterium clone (FJ202604)	Isolated from *Montastraea faveolata* kept in aquarium for 23 days	Sunagawa et al., 2009	
P.o_leaf_0BDVV (JF292441)	Leaf/Pujols	436	80	Uncultured gamma proteobacterium clone (DQ269096)	Isolated from surface of marine macro-alga *Delisea pulchra*	Longford et al., Unpublished	Sulfur-Oxidizing-Symbionts/γ-proteobacteria
			80	Uncultured gamma proteobacterium clone (FJ205337)	Isolated from deep marine sediments	Dong and Shao, Unpublished	
			80	Uncultured bacterium clone (EU491600 + EU491489 + EU491463)	Isolated from seafloor lavas from the East Pacific Rise	Santelli et al., 2008	
			80	Uncultured gamma proteobacterium (AB611274)	Isolated from abdominal setae of galatheid crab (*Shinkaia crosnieri*) at the Hatoma Knoll in the Okinawa Trough	Yoshida-Takashima et al., Unpublished	
			80	Uncultured gamma proteobacterium clone (AY534017)	Isolated from oxic surface sediments of eastern Mediterranean Sea	Polymenakou et al., 2005	
P.o_leaf_0BDVW (JF292442)	Leaf/Pujols	467	95	Sphingobacteriales bacterium (FJ952766)	Isolated from healthy tissue of coral *Montastrea annularis*	Rypien et al., 2010	*Flammeovirgaceae*/ Bacteroidetes
			95	*Flammeovirga aprica* (FJ917551)	Isolated from *Enteromorpha* (Green algae)	Zhang et al., Unpublished	
			95	*Flammeovirga sp.* (EF587968)	Isolated from natural subtidal biofilm	Huang et al., Unpublished	
			95	Uncultured bacterium clone (EF433127)	Isolated from *Favia* sp. mucus layer adjacent to Black Band mat	Barneah et al., 2007	
			95	*Flammeovirga sp.* (FN377813)	Bacteroidetes; Cytophagia; Cytophagales; Flammeovirgaceae; Flammeovirga.	Lu, Unpublished	
					Isolated from a marine gastropod mollusk *Haliotis diversicolor*		
P.o_leaf_0BDVX (JF292443)	Leaf/Pujols	447	87	*Flammeovirga apric*a (FJ917551)	Isolated from *Enteromorpha* (Green algae)	Zhang et al., Unpublished	*Flammeovirgaceae*/ Bacteroidetes
			86	Sphingobacteriales bacterium (FJ952766)	Isolated from healthy tissue of coral *Montastrea annularis*	Rypien et al., 2010	
			86	*Flammeovirga sp.* (EF587968)	Isolated from natural subtidal biofilm	Huang et al., Unpublished	
			86	Uncultured bacterium clone (EF433134)	Isolated from *Favia* sp. mucus layer adjacent to Black Band mat	Barneah et al., 2007	
			86	*Flammeovirga aprica* (FN554611)	Isolated from a marine gastropod mollusk *Haliotis diversicolor*	Zhao, Unpublished	
